# Racial, Ethnic, and Sex Differences in Social Risks and Social Needs Concordance Among Veterans

**DOI:** 10.1001/jamanetworkopen.2025.59892

**Published:** 2026-02-17

**Authors:** Lauren E. Russell, David A. Frank, Soumik Purkayastha, Jennifer L. McCoy, Sarah M. Leder, Joshua H. Gordon, Shane Lamba, Gregory T. Procario, Ernest M. Moy, Leslie R. M. Hausmann

**Affiliations:** 1 Office of Health Equity, Veterans Health Administration, Washington, DC; 2 Center for Healthcare Evaluation, Research, and Promotion, Veterans Affairs Pittsburgh Healthcare System, Pittsburgh, Pennsylvania; 3 Department of Biostatistics and Health Data Science, University of Pittsburgh School of Public Health, Pittsburgh, Pennsylvania; 4 Mental Illness Research, Education, and Clinical Center, Veterans Affairs Pittsburgh Healthcare System, Pittsburgh, Pennsylvania; 5 Department of Medicine, University of Pittsburgh School of Medicine, Pittsburgh, Pennsylvania

## Abstract

**Importance:**

Social screening initiatives often prioritize identifying social risks before offering support. The extent to which the emphasis on social risks contribute to overscreening and/or underdetection of needs, overall and across demographic subgroups, remains underexplored.

**Objective:**

To examine the agreement between self-reported social risks and needs for support and variance in risk-need concordance across racial, ethnic, and sex subgroups.

**Design, Setting, and Participants:**

This cross-sectional study examined responses to an online or mailed survey fielded between March 2 and May 9, 2023, to Veterans Health Administration patients who visited their primary care practitioner in January or February 2023. Survey weights were used to adjust for sampling frame and nonresponse. The data were analyzed between April 6 and December 15, 2025.

**Exposure:**

Intersection of race, ethnicity, and sex.

**Main Outcomes and Measures:**

Risk-need sensitivity, specificity, positive predictive value (PPV), negative predictive value (NPV), and concordance across 12 domains (paying for basics, obtaining adult caregiving, obtaining childcare, finding or keeping work, paying for food, finding or keeping housing, getting transportation, accessing the internet at home, feeling isolated, feeling lonely, getting legal assistance, and getting additional education or job training) were measured using age-adjusted relative risk ratios (ARRRs) of risk-need discordance (vs concordance), controlling for a family-wise error rate of .05.

**Results:**

The analytic sample included 6596 respondents, representing 937 003 veterans after weighting (unweighted number [weighted percentage]: aged <65 years, 2992 [48.5%]; aged ≥65 years, 3604 [51.5%]; 1088 identifying as Black female [4.1%], 1140 as Black male [19.4%], 939 as Hispanic female [1.6%], 1279 as Hispanic male [11.3%], 802 as White female [5.3%], and 1348 as White male [58.4%] race or ethnicity and sex). Across domains, risk-need sensitivity ranged from 42% (95% CI, 34%-49%) for housing to 99% (95% CI, 98%-99%) for loneliness, and specificity ranged from 69% (95% CI, 66%-71%) for loneliness to 98% (95% CI, 97%-99%) for childcare. The PPV ranged from 27% (95% CI, 22%-33%) for housing to 69% (95% CI, 63%-75%) for legal issues, and NPV ranged from 93% (95% CI, 91%-94%) for housing to 99% (95% CI, 99%-99%) for both childcare and loneliness. In age-adjusted models, compared with White male veterans, Black male veterans had a significantly higher likelihood of need-without-risk discordance for paying for basics (ARRR, 3.95; 95% CI, 1.80-8.64), housing (ARRR, 2.67; 95% CI, 1.59-4.48), and adult caregiving (ARRR, 2.13; 95% CI, 1.30-3.48). Additionally, compared with White male veterans, the likelihood of need-without-risk discordance for loneliness was higher among White female (ARRR, 14.02; 95% CI, 2.85-68.95), Hispanic female (ARRR, 10.87; 95% CI 2.31-51.25), and Hispanic male (ARRR, 8.08; 95% CI, 2.47-26.39) veterans. Differences for risk-without-need discordance were not significant.

**Conclusions and Relevance:**

This cross-sectional study of Veterans Health Administration patients found that social risk questions were better at identifying veterans without a social need than those who needed support and underdetected need for support among Black male veterans. Improved survey methods are essential to accurately detect needs in diverse populations.

## Key Points

**Question:**

Do self-reported social risks capture need for support overall, and does risk-need concordance vary across intersecting racial and ethnic identities and sex?

**Findings:**

In this cross-sectional study of 6596 primary care patients representing 937 003 veterans after weighting, Black male veterans had a significantly higher odds of reporting social needs without accompanying social risks in several domains. No differences among racial, ethnic, and sex subgroups were found for reporting social risks without social needs, although these occurred at a higher rate overall.

**Meaning:**

These findings suggest that overall agreement between self-reported social risks and need for support may be modest at best and that social risk questions may underdetect the need for support more often among Black male veterans.

## Teaser

This cross-sectional study examines the agreement between self-reported social risks and needs for support and variance in risk-need concordance across racial, ethnic, and sex subgroups of US veterans.

## Introduction

Policymakers, professional organizations, and payers have advocated for expanding processes to identify social risks and address health-related social needs (hereafter, social needs), the midstream manifestations of social determinants or drivers of health, in the context of routine clinical care.^1,2,3,4^ Though often used interchangeably, social risks and social needs are distinct. Social risks are individual-level conditions associated with poor health, such as housing instability and unemployment.^3,4,5^ In contrast, social needs reflect a person’s perceptions that they have a need and are interested in addressing it.^5^

In the Veterans Health Administration (VHA), veterans are routinely screened for social risks through national clinical reminders for housing instability,^6^ food insecurity,^7^ and intimate partner violence.^8^ The VHA also screens for social risks more broadly through the Assessing Circumstances and Offering Resources for Needs social risks screening and referral intervention.^9^ Positive responses to the clinical reminders and Assessing Circumstances and Offering Resources for Needs prompt follow-up with staff (eg, nurses, social workers, peer specialists) to further assess whether any reported risks are perceived by the patient as a social need for which they would like to receive assistance.^10^

Studies have found notable dropoffs between reported social risks and subsequent reporting of need for support.^11,12,13,14,15,16,17,18,19,20,21,22^ Patients may also report social needs even if they screen negative for the corresponding social risk.^15,19,21^ Understanding discordance between reporting social risks and social needs (ie, risk-need discordance) is important to understand overdetection or underdetection in need for support, maximize the use of constrained and finite resources within health care systems, and minimize the number of patients whose health outcomes are impacted by their unmet social needs. However, few studies have systematically analyzed agreement between self-reported social risks and needs, and even fewer have compared risk-need discordance across demographic subgroups who may have different risks and needs due to the unequal distribution of social determinants or drivers of health.

To address these gaps, we conducted a survey of social risks and social needs among a nationally representative sample of veterans receiving VHA primary care services. The goals of the study were to examine (1) agreement (as defined by sensitivity, specificity, positive predictive value [PPV], and negative predictive value [NPV]) between self-reported social risks and need for support and (2) how risk-need concordance may vary across racial, ethnic, and sex subgroups.

## Methods

This cross-sectional study was deemed exempt from review and informed consent by the Veterans Affairs Pittsburgh Healthcare System Institutional Review Board because it used deidentified data collected for nonresearch purposes. The study followed the Strengthening the Reporting of Observational Studies in Epidemiology (STROBE) reporting guideline for cross-sectional studies.^23^

### Study Design and Survey Administration

We conducted a cross-sectional analysis of data collected via the VHA Your Recent Visit survey, which assesses patient experiences with a recent medical appointment.^24^ As detailed elsewhere,^25^ veterans with a VHA primary care visit in January or February 2023 were invited to complete the survey by mail or email between March 2 and May 9, 2023. We oversampled White female patients and Black and Hispanic male and female patients to reach statistical power to assess differences across strata defined by race, ethnicity, and sex.^26,27^ Though race and ethnicity are social constructs and imperfect proxies to assess systematic differences in experiences of different groups, they are used in our analyses to understand how social risks and needs differ across intersecting marginalized identities.^28^

### Primary Exposure: Intersection of Race, Ethnicity, and Sex

Groups were oversampled to yield approximately 900 respondents each from 6 racial, ethnic, and sex groups: Black female, Black male, Hispanic female, Hispanic male, White female, and White male veterans.^29^ Race was based on responses to a survey question asking respondents to select all that apply from the following options: American Indian or Alaska Native, Asian, Black or African American, Native Hawaiian or Other Pacific Islander, and White. Ethnicity was assessed based on the question, “Are you of Hispanic or Latino origin or descent? (yes or no).” Sex was assessed based on the question, “What sex is listed on your birth certificate? (female or male).” For respondents with missing self-reported data (unweighted number [weighted percentage]), race (502 [4.6%]), ethnicity (246 [3.4%]), and sex (90 [1.4%]) were ascertained from the VHA Corporate Data Warehouse. Race and ethnicity in the Corporate Data Warehouse are mostly self-reported responses collected during VHA enrollment or scheduling for services.^29^ Respondents who self-identified as Hispanic were categorized as Hispanic, regardless of self-reported race. Non-Hispanic Black and non-Hispanic White respondents are subsequently referred to as Black and White, respectively. Due to small sample sizes, we excluded respondents who self-identified as non-Hispanic American Indian or Alaska Native, Asian, and Native Hawaiian or Other Pacific Islander, as well as veterans who selected multiple races.

### Measures: Social Risks and Social Needs

Social risk and social needs items were developed following the protocol for adding new items to VHA patient experience surveys.^24^ Self-reported social risks and social needs were assessed for 12 domains (eAppendix in Supplement 1): (1) paying for basics such as food, housing, medical care, and heating; (2) obtaining adult caregiving for yourself or others; (3) obtaining childcare; (4) finding or keeping work; (5) paying for food; (6) getting or maintaining housing; (7) getting transportation for basic needs such as medical care or grocery shopping; (8) accessing the internet at home; (9) feeling socially isolated; (10) feeling lonely; (11) getting assistance with legal issues; and (12) getting additional education or job training.

For most social risks, frequency of experiencing the risk over the past 6 months was assessed using a Likert scale consisting of never, sometimes, usually, or always. The domains of adult caregiving, childcare, and work also included an option indicating that the risk was not applicable (eg, not working or looking for work); this response was combined with never for analyses. Legal risk was assessed using a yes or no response to, “In the past 6 months, have you had any legal issues you needed help with?” A positive risk assessment was defined as any response other than never, not applicable, or no, except for 2 reverse-coded domains (ie, accessing the internet at home, getting or maintaining housing), for which any response other than always indicated positive risk.

Social needs questions were presented in a grid prefaced by, “In the past 6 months, did you need support with any of the following?” For each domain, respondents could select no support needed, needed support and got it, or needed support but did not get it. For analyses, respondents who selected needed support and got it or needed support but did not get it were considered positive for a social need. Respondents with missing data on all social risk and needs items were excluded.

### Outcomes: Measures of Agreement Between Social Risks and Social Needs

Positive screens for each social need were used as the criterion standard by which to compare the corresponding social risk, given that self-reported need for support serves as the best available proxy for a patient’s perceived need and desire for seeking support to address this need.^5^ In this study, sensitivity refers to the ability of a risk question to identify participants with a corresponding need for support. Specificity refers to the ability of a risk question to correctly identify participants without a corresponding need for support. The PPV and NPV reflect the probability that participants who screened positive or negative for the risk either did or did not report a corresponding need, respectively.^30^ The PPVs and NPVs are reported as percentages.

A 3-level variable to assess risk-need concordance was constructed for each domain. Screening positive or negative on the corresponding risk and needs questions for a given domain was defined as concordant. Screening positive for a risk but not reporting the corresponding need was defined as risk-without-need discordance. Screening negative for a risk but reporting the corresponding need was defined as need-without-risk discordance.

### Statistical Analysis

The data were analyzed between April 6 and December 15, 2025. Design weights and nonresponse weights were applied to account for the sampling frame and differential response rates across age and racial, ethnic, and sex subgroups. Unweighted numbers of participants and weighted percentages are reported. Rao-Scott second-order corrected Pearson χ^2^ tests were used to test differences in demographics.

Sensitivity, specificity, PPV, and NPV are reported for each domain in the overall sample. Unadjusted rates of concordant, risk-without-need, and need-without-risk responses for each domain are presented overall and across groups. We developed multinomial logistic regression models for each domain to examine the association between concordance (concordant [reference] vs risk without need and concordant vs need without risk) and race, ethnicity, and sex. We calculated age-adjusted relative risk ratios (ARRRs), using the largest categories as the reference groups (ie, White male veterans and aged 65-74 years). Age category was self-reported on the survey and supplemented with Corporate Data Warehouse data when missing (90 [1.2%]). To adjust for multiple comparisons, *P* values from each multinomial model were adjusted to control for a family-wise error rate of .05 according to the Holm method.^31^ Adjusted *P* < .05 was considered statistically significant. Analyses and figure creation were performed using the 64-bit version of R, version 4.3.2 (R Foundation for Statistical Computing); multinomial models were used to account for survey weights using the survey package in R.^32^

## Results

### Sample Characteristics

**Figure 1.**
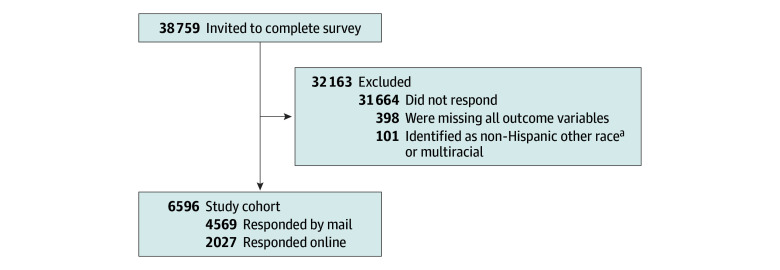
Flowchart for the Analytic Cohort ^a^Included American Indian or Alaska Native, Asian, and Native Hawaiian or Other Pacific Islander.

Of the 38 759 patients invited to participate, the analytic sample included 6596 respondents (17.0%) (Figure 1), representing 937 003 veterans after weighting (unweighted number [weighted percentage]: aged <65 years, 2992 [48.5%]; aged ≥65 years, 3604 [51.5%], 1088 identifying as Black female [4.1%], 1140 as Black male [19.4%], 939 as Hispanic female [1.6%], 1279 as Hispanic male [11.3%], 802 as White female [5.3%], and 1348 as White male [58.4%] race or ethnicity and sex) (Table 1). Respondents were more likely to be White, male, and older than nonrespondents (eTable 1 in Supplement 1).

**Table 1.** Age Distribution of Survey Respondents Overall and by Race, Ethnicity, and Sex

Characteristic	Respondents, unweighted No. (weighted %)							*P* value
	Overall	Female			Male			
		Black	Hispanic	White	Black	Hispanic	White	
Unweighted No. of respondents^a^	6596	1088	939	802	1140	1279	1348	NA
Weighted No. of respondents	937 003	38 104	14 800	49 373	181 755	106 228	546 742	NA
Age category, y								
18-44^b^	595 (16.4)	127 (34.8)	242 (61.5)	77 (32.2)	23 (10.5)	93 (35.6)	33 (10.6)	<.001
45-54	791 (12.7)	185 (19.6)	212 (16.6)	131 (21.9)	82 (14.6)	113 (11.9)	68 (10.8)	
55-64	1606 (19.4)	457 (31.1)	251 (13.5)	258 (25.3)	266 (27.4)	217 (17.2)	157 (16.0)	
65-74	2008 (25.5)	283 (13.2)	192 (7.1)	241 (15.3)	451 (30.5)	414 (18.7)	427 (27.4)	
≥75	1596 (26.0)	36 (1.3)	42 (1.3)	95 (5.2)	318 (17.0)	442 (16.6)	663 (35.1)	

Abbreviation: NA, not applicable.

^a^ Missing self-reported data were supplemented with administrative data for 502 participants missing race, 246 missing ethnicity, 90 missing sex, and 90 missing age.

^b^ For analyses, response categories 18 to 24 years (n = 11), 25 to 34 years (n = 117), and 35 to 44 years (n = 467) were combined due to small numbers.

### Sensitivity, Specificity, PPV, and NPV

Across domains, sensitivity (ie, the percentage of patients who screened positive for a risk among those who reported needing support for the same domain) ranged from 42% (95% CI, 34%-49%) for housing to 99% (95% CI, 98%-99%) for loneliness, with a median of 79% (IQR, 67%-86%) (Table 2). Specificity (ie, the percentage of patients who screened negative for a risk among those who reported not needing support for the same domain) ranged from 69% (95% CI, 66%-71%) for loneliness to 98% (95% CI, 97%-99%) for childcare, with a median of 88% (IQR, 76%-92%). The PPV (ie, percentage of patients who reported a need for support among those who screened positive for risk in the same domain) ranged from 27% (95% CI, 22%-33%) for housing to 69% (95% CI, 63%-75%) for legal issues, with a median of 47% (IQR, 41%-55%). The NPV (ie, percentage of patients who reported no need for support among those who screened negative for risk in the same domain) was higher overall, ranging from 93% (95% CI, 91%-94%) for housing to 99% (95% CI, 99%-99%) for both childcare and loneliness, with a median of 97% (IQR, 96%-97%).

**Table 2.** Sensitivity, Specificity, PPV, and NPV of Social Risk Domains

Social domain	% (95% CI)			
	Sensitivity	Specificity	PPV	NPV
Feeling lonely	99 (98-99)	69 (66-71)	56 (52-59)	99 (99-99)
Feeling socially isolated	92 (88-95)	74 (71-76)	55 (51-59)	96 (94-98)
Paying for basics	89 (86-92)	76 (74-78)	46 (42-50)	97 (96-98)
Paying for food	86 (81-89)	89 (87-91)	60 (55-65)	97 (96-98)
Accessing the internet at home	82 (77-86)	71 (69-74)	30 (27-34)	96 (95-97)
Assistance with legal issues	78 (73-83)	95 (94-96)	69 (63-75)	97 (96-98)
Transportation for basic needs	69 (62-75)	95 (94-96)	63 (57-69)	96 (95-97)
Adult caregiving for self or others	54 (47-61)	92 (91-94)	48 (41-55)	94 (93-95)
Getting additional education or job training	79 (70-86	86 (84-88)	41 (35-47)	97 (95-98)
Getting or maintaining housing	42 (34-49)	87 (85-89)	27 (22-33)	93 (91-94)
Finding or keeping work	79 (72-85)	90 (88-92)	45 (38-53)	98 (97-98)
Obtaining childcare	68 (55-79)	98 (97-99)	46 (33-60)	99 (99-99)

Abbreviations: NPV, negative predictive value; PPV, positive predictive value.

### Social Risk-Need Concordance Overall and by Race, Ethnicity, and Sex

The unadjusted concordance between self-reported risks and needs ranged from 72.6% for internet access to 97.2% for childcare (Table 3). The percentage of risk-without-need discordance ranged from 2.0% for childcare to 25.0% for internet access. The percentage of need-without-risk discordance was generally lower, ranging from 0.4% for loneliness to 5.9% for housing. Age-adjusted multinomial models using concordant survey responses as the reference outcome showed no significant racial, ethnic, and sex differences in risk-without-need discordance after adjusting *P* values for multiple comparisons (eTable 2 in Supplement 1).

**Table 3.** Concordance of Social Risks and Social Needs Overall and by Race, Ethnicity, and Sex

Characteristic^a^	Respondents, weighted %						
	Overall	Female			Male		
		Black	Hispanic	White	Black	Hispanic	White
Feeling lonely							
Concordant	77.3	77.7	78.6	77.0	80.8	77.8	76.1
Risk without need	22.3	21.9	20.5	21.7	18.7	21.2	23.8
Need without risk	0.4	0.4	0.8	1.3	0.5	0.9	0.2
Feeling socially isolated							
Concordant	78.4	77.0	73.4	77.1	79.3	76.7	78.8
Risk without need	19.6	19.4	24.9	21.7	17.2	21.5	19.7
Need without risk	2.0	3.6	1.8	1.2	3.5	1.8	1.5
Paying for basics							
Concordant	78.4	82.0	84.3	84.6	77.3	75.3	78.4
Risk without need	19.6	15.0	12.7	14.6	18.2	22.8	20.4
Need without risk	2.0	3.1	2.9	0.8	4.5	1.9	1.2
Paying for food							
Concordant	88.6	87.8	89.3	89.9	85.0	86.5	90.1
Risk without need	9.1	9.2	8.0	7.8	10.6	10.7	8.4
Need without risk	2.3	3.0	2.7	2.3	4.4	2.8	1.4
Accessing the internet at home							
Concordant	72.6	76.9	74.6	75.7	71.0	67.7	73.5
Risk without need	25.0	20.0	22.4	23.0	25.8	30.2	24.3
Need without risk	2.4	3.1	3.0	1.3	3.2	2.1	2.2
Getting assistance with legal issues							
Concordant	92.9	91.0	94.5	96.4	91.5	94.2	92.8
Risk without need	4.4	4.2	2.5	2.7	4.9	2.0	4.9
Need without risk	2.7	4.8	3.0	0.9	3.5	3.8	2.3
Transportation for basic needs							
Concordant	91.8	90.4	87.5	95.0	88.4	91.5	92.8
Risk without need	4.7	4.9	8.2	3.6	5.5	4.4	4.4
Need without risk	3.6	4.7	4.3	1.4	6.1	4.2	2.7
Adult caregiving for self or others							
Concordant	87.9	90.0	90.3	92.0	84.6	84.9	89.0
Risk without need	6.8	5.0	4.3	4.5	7.2	9.0	6.7
Need without risk	5.3	5.0	5.3	3.5	8.2	6.1	4.3
Getting additional education or job training							
Concordant	84.9	79.6	75.9	85.1	84.3	76.0	87.5
Risk without need	12.7	14.9	21.3	12.1	13.0	21.6	10.6
Need without risk	2.3	5.5	2.8	2.9	2.7	2.5	1.9
Getting or maintaining housing							
Concordant	82.7	80.9	77.5	85.3	76.9	75.4	86.1
Risk without need	11.3	10.7	9.5	8.8	13.2	15.3	10.3
Need without risk	5.9	8.4	13.0	5.9	9.9	9.3	3.6
Finding or keeping work							
Concordant	89.3	88.9	84.7	89.0	90.1	80.9	90.8
Risk without need	8.8	10.2	13.2	8.4	7.4	16.6	7.6
Need without risk	1.9	1.0	2.0	2.7	2.6	2.6	1.6
Obtaining childcare							
Concordant	97.2	97.6	96.2	97.5	96.5	94.1	98.0
Risk without need	2.0	1.4	1.9	1.8	2.5	4.3	1.4
Need without risk	0.8	1.1	1.8	0.7	1.0	1.6	0.5

^a^ A concordant response is defined as having the same positive or negative response to both the risk and need questions for a given domain. A risk-without-need response is defined as screening positive for a risk but negative for the corresponding need. A need-without-risk response is defined as screening positive for a need but negative for the corresponding risk.

**Figure 2.**
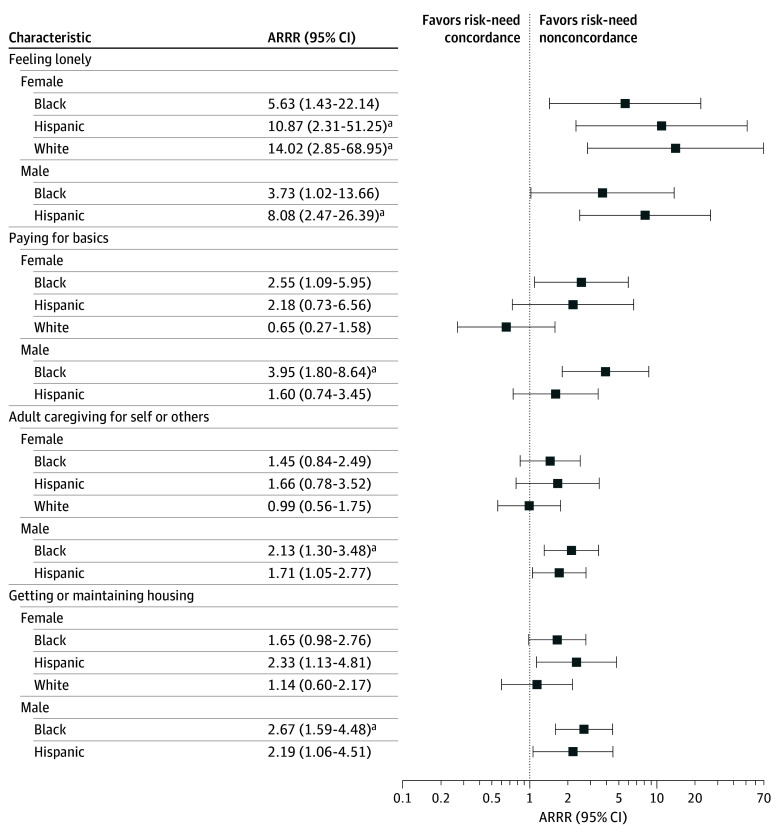
Racial, Ethnic, and Sex Differences in Age-Adjusted Odds of a Need-Without-Risk Screening Models were adjusted for age group. The reference was White male veterans. Domains with significant racial, ethnic, and sex differences were selected for visualization due to space limitations. eTable 2 in Supplement 1 provides the full results. ARRR indicates adjusted relative risk ratio. ^a^Results were significant after adjusting for multiple comparisons according to the Holm method.

There were significant age-adjusted racial, ethnic, and sex differences in need-without-risk discordance in 4 domains (Figure 2; eTable 2 in Supplement 1). Compared with White male respondents, Black male respondents had a higher odds of need-without-risk discordance over a concordant response in the domains of paying for basics (ARRR, 3.95; 95% CI, 1.80-8.64), housing (ARRR, 2.67; 95% CI, 1.59-4.48), and adult caregiving (ARRR, 2.13; 95% CI, 1.30-3.48). In the domain of feeling lonely, compared with White male respondents, White female (ARRR, 14.02; 95% CI, 2.85-68.95), Hispanic female (ARRR, 10.87; 95% CI 2.31-51.25), and Hispanic male (ARRR, 8.08; 95% CI, 2.47-26.39) respondents had a higher odds of need-without-risk discordance.

## Discussion

This cross-sectional study uncovered detailed information regarding agreement between self-reported social risks and social needs in a diverse sample of veterans receiving care from the VHA. Overall, measures of sensitivity, specificity, PPV, and NPV showed that risk questions were better at identifying individuals without a social need than those who had a need for support. In addition, risk-need concordance was moderate to high across 12 domains, and risk-without-need discordance was more common than need-without-risk discordance. Importantly, we identified significant variation by race, ethnicity, and sex in need-without-risk discordance in 4 domains (paying for basics, housing, adult caregiving, and feeling lonely), mostly among Black male respondents. In contrast, we found no significant differences in the likelihood of risk-without-need discordance. Arguably, need-without-risk discordance may be more clinically significant because further screening or the provision of resources in traditional point-of-care social screening programs often depends on the initial identification of social risks.^33,34^ Our findings suggest that Black male veterans may be more likely to have their needs go undetected when screening instruments focus on identifying social risks.

While several studies have found higher rates of needs reported among Black^14,16,19,25^ and Hispanic^14^ individuals, little research has evaluated racial, ethnic, or sex differences in patterns of risk-need discordance. One exception includes findings from a large Medicaid accountable care organization in which Black race and Hispanic ethnicity, but not sex, were associated with need-without-risk discordance.^15^ Further research is needed to understand reasons for higher rates of need-without-risk discordance observed among Black male veterans in this study. Possible reasons may include differences in question interpretation or use of response options; unwillingness to disclose risks due to independence and self-reliance; mistrust toward medical professionals or health systems, which could lead to seeking support outside of these individuals and institutions; emotional stoicism; or stigma.^35,36,37,38^ Regardless of the underlying drivers of need-without-risk discordance among Black male veterans, existing inequities could be further exacerbated by relying on positive social risk screening as a precursor for offering support.

Consistent with prior studies,^11,12,13,14,15,16,17,18,19,20,21,22^ we found that risk-without-need discordance was more common than need-without-risk discordance. Relatively high prevalence of risk-without-need discordance may place an undue burden on the patient’s care team members, strain finite resources within health care systems, and limit time available to focus on patient’s acute care needs and preferences. Patients may report a social risk without a corresponding need for support for numerous reasons. For example, patients may already be receiving support and/or do not feel as though they need or want help from the health care system.^12,39^ They may also be uncertain about the type of assistance available if they do express a desire for support and/or have had prior negative experiences with social service resources or referrals.^12,39,40,41^ The ordering of risk and need questions may also influence concordance.^19^

By examining multiple measures of agreement (sensitivity, specificity, PPV, and NPV), we found that the social risk questions were better at identifying individuals without social needs than those who needed support (ie, true-negative findings were better identified than true-positive findings). For example, PPVs were 63% or less for all domains, indicating a low to moderate probability that the risk questions would identify those with a need for support, depending on the domain. In contrast, NPVs were all more than 90%, indicating a high probability that the risk questions would correctly identify individuals without a need for support. Unfortunately, there is no established criterion standard or threshold for sensitivity and specificity of social screening instruments because of the variability and prevalence of social risks and social needs, lack of standardized screening questions or measures, screening objectives, and available resources within and across health care settings.^12,38,42^ Efforts to find optimal methods to identify individuals with health-related social factors have found that screening questionnaires and automated approaches (eg, natural language processing of clinical notes) all have limitations and may underestimate true social burden if used in isolation.^43^

The distinction between social risks and social needs we found should not be interpreted as an endorsement of favoring one method of social screening over the other. Instead, the findings reaffirm the importance of assessing both social risks and social needs given their shared and unique benefits. Optimal practices for screening may vary based on patient preferences, prevalence of social risks and social needs among patients, available resources (eg, staffing capacity, supportive services and interventions within the health care system and community), technological infrastructure (eg, electronic self-administered screening vs staff-administered screening), and the health system’s goals.^3,5^ For example, research has indicated that screening for social risks may not need to be done frequently due to their stability over time, while social needs screening may be better suited to point-of-care initiatives in which immediate support can be offered.^44^ There remains differing viewpoints among experts in the field on a variety of aspects of social screening, including the optimal timing and frequency of screening; the circumstances in which screening is used; and, to some extent, the overall purpose of screening for social risks or social needs. Garg et al^45^ argued that, “screening for any condition in isolation without the capacity to ensure referral and linkage to appropriate treatment is ineffective and, arguably, unethical”; meanwhile, Byhoff and Gottlieb^46^ noted that there are several benefits to social screening initiatives irrespective of the joint screening and referral pathway. Our findings add to this ongoing debate by offering evidence that solely focusing on the identification of social risks may systematically underdetect need for support, especially among Black male veterans.

### Limitations

Our study had some limitations. While our sampling strategy allowed for evaluating differences across targeted racial, ethnic, and sex groups, we did not have sufficient data to extend analyses to veterans who reported being of multiple races or from numerically small populations. Additionally, our sample consisted of veterans who received VHA primary care services and responded to a postvisit survey, which limits generalizability to nonveterans and veterans not using VHA health care. Our study was also limited to 1 screening tool. Similar studies in other health systems may produce different results, depending on the properties of the screening tool used and the patient population screened.

## Conclusions

This cross-sectional study found racial, ethnic, and sex differences in measures of concordance of self-reported social risks and social needs among veterans receiving VHA primary care. Our findings underscore the complexity of identifying patients with a social need for which they are interested in receiving supportive services to address it. Our findings also suggest that self-reported social risks are conceptually distinct from, and do not completely align with, self-reported social needs. Further evaluation of these 2 concepts, how they vary across populations, and their association with the provision of resources within health systems is vital to developing effective social-care interventions that meet the needs of all patients.

## Notes

Supplement 1. **eAppendix.** Social Risk and Social Need Questionnaire **eTable 1.** Comparison of Survey Responders and Nonresponders **eTable 2.** Racial, Ethnic, and Sex Differences in Age-Adjusted Odds of Discordant Screening Responses

Supplement 2.
Data Sharing Statement
