# Supplementary material for: Racial, Ethnic, and Sex Differences in Social Risks and Social Needs Concordance Among Veterans
Source: JAMA Netw Open. 2026 Feb 17;9(2):e2559892. doi: 10.1001/jamanetworkopen.2025.59892 (PMC12914491; doi:10.1001/jamanetworkopen.2025.59892)
Supplement: Supplement 1. — eAppendix. Social Risk and Social Need Questionnaire eTable 1. Comparison of Survey Responders and Nonresponders eTable 2. Racial, Ethnic, and Sex Differences in Age-Adjusted Odds of Discordant Screening Responses [file jamanetwopen-e2559892-s001.pdf]

Supplemental Online Content

Russell LE, Frank DA, Purkayastha S, et al. Racial, ethnic, and sex differences in social risks and social needs concordance among veterans. *JAMA Netw Open*. 2026;9(2):e2559892. doi:10.1001/jamanetworkopen.2025.59892

eAppendix. Social Risk and Social Need Questionnaire

eTable 1. Comparison of Survey Responders and Nonresponders

eTable 2. Racial, Ethnic, and Sex Differences in Age-Adjusted Odds of Discordant Screening Responses

This supplemental material has been provided by the authors to give readers additional information about their work.

© 2026 Russell LE et al. *JAMA Network Open*.

eAppendix. Social Risk and Social Need Questionnaire

The following questions ask about social circumstances that can influence health. Your answers will help the VA develop partnerships with other organizations to help meet Veterans' needs.

Your Social Circumstances[a]

1. In the past 6 months, how often was it **hard for you to pay for the very basics** like food, housing, medical care, and heating?
   - ☐ Never
   - ☐ Sometimes
   - ☐ Usually
   - ☐ Always

2. In the past 6 months, how often were you **unable** to get assistance with adult caregiving for yourself or others when it was **really needed**?
   - ☐ Never
   - ☐ Sometimes
   - ☐ Usually
   - ☐ Always
   - ☐ No adult caregiving needs in the past 6 months

3. In the past 6 months, how often were you or any family members you live with **unable** to get childcare when it was **really needed**?
   - ☐ Never
   - ☐ Sometimes
   - ☐ Usually
   - ☐ Always
   - ☐ No childcare needs in the past 6 months

4. In the past 6 months, how often were you worried about finding or keeping work?
   - ☐ Never
   - ☐ Sometimes
   - ☐ Usually
   - ☐ Always
   - ☐ Not working or looking for work in the past 6 months

5. In the past 6 months, how often were you worried that your food would run out before you got money to buy more?
   - ☐ Never
   - ☐ Sometimes
   - ☐ Often

© 2026 Russell LE et al. *JAMA Network Open*.

- ☐ Always

6. In the past 6 months, how often did you live in stable housing that you own, rent, or stay in as part of a household?[b]
   - ☐ Never
   - ☐ Sometimes
   - ☐ Usually
   - ☐ Always

7. In the past 6 months, how often has lack of transportation kept you from medical appointments or from doing things needed for daily living?
   - ☐ Never
   - ☐ Sometimes
   - ☐ Usually
   - ☐ Always

8. In the past 6 months, how often did you have access to affordable and reliable internet at home?[b]
   - ☐ Never
   - ☐ Sometimes
   - ☐ Usually
   - ☐ Always

9. In the past 6 months, how often did you experience a lack of social connections with others or feel socially isolated?
   - ☐ Never
   - ☐ Sometimes
   - ☐ Usually
   - ☐ Always

10. In the past 6 months, how often did you feel lonely?
    - ☐ Never
    - ☐ Sometimes
    - ☐ Usually
    - ☐ Always

11. In the past 6 months, have you had any legal issues you needed help with?
    - ☐ Yes
    - ☐ No

12. In the past 6 months, how often did you feel that lack of education and/or job training was holding you back from making enough money?
    - ☐ Never
    - ☐ Sometimes
    - ☐ Usually
    - ☐ Always

© 2026 Russell LE et al. *JAMA Network Open*.

13. In the past 6 months, did you **need support** with any of the following:

	No support needed	Needed support and got it	Needed support but did not get it
Paying for basics like food, housing, medical care, and heating			
Obtaining adult caregiving for yourself or others			
Obtaining childcare			
Finding or keeping work			
Paying for food			
Getting or maintaining housing			
Getting transportation for basic needs like medical care or grocery shopping			
Accessing the internet at home			
Feeling socially isolated			
Feeling lonely			
Getting assistance with legal issues			
Getting additional education or job training			

[a]A positive risk assessment was defined as any response other than “not applicable” or “Never”, except for two reverse-coded domains (i.e., accessing internet at home; getting or maintaining housing), for which any response other than “always” indicated positive risk. “Yes” indicated a positive response for legal issues. For analyses, those who selected “needed support and got it” or “needed support but did not get it” were treated as a positive screen for social need.

[b] These response items were reverse-coded such that any response other than “always” indicated positive risk.

© 2026 Russell LE et al. *JAMA Network Open*.

eTable 1. Comparison of Survey Responders and Nonresponders

Characteristic[a]	Survey Recipients		
	Non-Responder	Responder	P value
No. of participants	31,664	7,095	
Sampled strata			
Black Female	7801 (24.6)	1199 (16.9)	
Hispanic Female	7850 (24.8)	1059 (14.9)	<0.001
White Female	3549 (11.2)	851 (12.0)	
Black Male	4778 (15.1)	1222 (17.2)	
Hispanic Male	4660 (14.7)	1390 (19.6)	
White Male	3026 (9.6)	1374 (19.4)	
Age group			
18 - 34	4785 (15.1)	161 (2.3)	<0.001
35 - 44	7105 (22.4)	546 (7.7)	
45 - 54	6319 (20.0)	901 (12.7)	
55 - 64	6459 (20.4)	1736 (24.5)	
65 to 74	4669 (14.8)	2121 (29.9)	
75 or older	2327 (7.4)	1630 (23.0)	
Type of visit[b]			
By phone	5659 (17.9)	1171 (16.5)	<0.001
Video visit	2630 (8.3)	429 (6.1)	
In clinician's office	23365 (73.8)	5488 (77.4)	

[a] Data were extracted from the administrative record and are expressed as No.(%) unless otherwise indicated.
[b] Based on the visit type associated with the primary care visit between January and February 2023 connected with this survey.

© 2026 Russell LE et al. *JAMA Network Open*.

eTable 2. Racial, Ethnic, and Sex Differences in Age-Adjusted Odds of Discordant Screening Responses

Domain	Characteristic[a]	aRRR [95% CI]	P value	Adjusted P value[b]
**Feeling Lonely**	**Need-Without-Risk**			
	Black Female	5.63 [1.43 to 22.14]	0.01	0.09
	Hispanic Female	10.87 [2.31 to 51.25]	0.003	0.02
	White Female	14.02 [2.85 to 68.95]	0.001	0.01
	Black Male	3.73 [1.02 to 13.66]	0.05	0.24
	Hispanic Male	8.08 [2.47 to 26.39]	<0.001	0.005
	White Male	1 [Reference]		
	18 to 44	0.25 [0.05 to 1.38]	0.11	
	45 to 54	0.28 [0.10 to 0.78]	0.02	
	55 to 64	0.08 [0.02 to 0.27]	<0.001	
	65 to 74	1 [Reference]		
	75 or older	0.72 [0.29 to 1.80]	0.48	
	**Risk-Without-Need**			
	Black Female	0.81 [0.57 to 1.16]	0.25	0.75
	Hispanic Female	0.67 [0.40 to 1.14]	0.14	0.55
	White Female	0.82 [0.57 to 1.19]	0.30	0.75
	Black Male	0.73 [0.56 to 0.96]	0.02	0.14
	Hispanic Male	0.78 [0.50 to 1.22]	0.27	0.75
	White Male	1 [Reference]		
	18 to 44	1.36 [0.82 to 2.27]	0.23	
	45 to 54	0.87 [0.57 to 1.32]	0.52	
	55 to 64	0.86 [0.64 to 1.16]	0.32	
	65 to 74	1 [Reference]		
	75 or older	0.85 [0.66 to 1.09]	0.20	
**Feeling socially isolated**	**Need-Without-Risk**			
	Black Female	1.43 [0.33 to 6.18]	0.64	1.00
	Hispanic Female	0.48 [0.09 to 2.63]	0.40	1.00
	White Female	0.49 [0.10 to 2.29]	0.36	1.00
	Black Male	2.41 [0.75 to 7.78]	0.14	1.00
	Hispanic Male	0.70 [0.16 to 3.11]	0.64	1.00
	White Male	1 [Reference]		
	18 to 44	7.58 [1.78 to 32.39]	0.006	
	45 to 54	1.52 [0.56 to 4.11]	0.41	
	55 to 64	1.32 [0.68 to 2.56]	0.41	
	65 to 74	1 [Reference]		
	75 or older	1.65 [0.85 to 3.17]	0.14	
	**Risk-Without-Need**			

© 2026 Russell LE et al. *JAMA Network Open*.

	Black Female	0.82 [0.56 to 1.21]	0.32	1.00
	Hispanic Female	0.91 [0.52 to 1.60]	0.76	1.00
	White Female	0.93 [0.63 to 1.38]	0.73	1.00
	Black Male	0.85 [0.63 to 1.15]	0.30	1.00
	Hispanic Male	0.91 [0.57 to 1.45]	0.69	1.00
	White Male	1 [Reference]		
	18 to 44	1.81 [1.08 to 3.04]	0.03	
	45 to 54	0.90 [0.59 to 1.38]	0.64	
	55 to 64	0.86 [0.63 to 1.17]	0.34	
	65 to 74	1 [Reference]		
	75 or older	0.79 [0.60 to 1.03]	0.08	
**Paying for basics**	**Need-Without-Risk**			
	Black Female	2.55 [1.09 to 5.95]	0.03	0.27
	Hispanic Female	2.18 [0.73 to 6.56]	0.16	0.82
	White Female	0.65 [0.27 to 1.58]	0.34	0.91
	Black Male	3.95 [1.80 to 8.64]	<0.001	0.006
	Hispanic Male	1.60 [0.74 to 3.45]	0.23	0.91
	White Male	1 [Reference]		
	18 to 44	0.91 [0.20 to 4.03]	0.90	
	45 to 54	0.43 [0.18 to 1.01]	0.05	
	55 to 64	0.60 [0.32 to 1.14]	0.12	
	65 to 74	1 [Reference]		
	75 or older	0.65 [0.33 to 1.30]	0.23	
	**Risk-Without-Need**			
	Black Female	0.71 [0.49 to 1.03]	0.07	0.48
	Hispanic Female	0.65 [0.40 to 1.06]	0.09	0.53
	White Female	0.68 [0.46 to 1.00]	0.05	0.40
	Black Male	0.87 [0.66 to 1.14]	0.32	0.91
	Hispanic Male	1.22 [0.84 to 1.77]	0.30	0.91
	White Male	1 [Reference]		
	18 to 44	0.75 [0.42 to 1.34]	0.33	
	45 to 54	0.86 [0.55 to 1.32]	0.48	
	55 to 64	1.24 [0.91 to 1.69]	0.18	
	65 to 74	1 [Reference]		
	75 or older	0.91 [0.70 to 1.18]	0.46	
**Paying for food**	**Need-Without-Risk**			
	Black Female	1.72 [0.78 to 3.79]	0.18	1.00
	Hispanic Female	1.56 [0.57 to 4.24]	0.38	1.00
	White Female	1.33 [0.46 to 3.85]	0.60	1.00
	Black Male	2.84 [1.20 to 6.71]	0.02	0.17
	Hispanic Male	1.78 [0.81 to 3.95]	0.15	1.00

© 2026 Russell LE et al. *JAMA Network Open*.

	White Male	1 [Reference]		
	18 to 44	0.94 [0.23 to 3.75]	0.93	
	45 to 54	0.77 [0.22 to 2.74]	0.69	
	55 to 64	1.05 [0.59 to 1.88]	0.86	
	65 to 74	1 [Reference]		
	75 or older	0.39 [0.18 to 0.84]	0.02	
	**Risk-Without-Need**			
	Black Female	0.92 [0.55 to 1.54]	0.74	1.00
	Hispanic Female	0.89 [0.50 to 1.60]	0.70	1.00
	White Female	0.78 [0.46 to 1.33]	0.36	1.00
	Black Male	1.16 [0.80 to 1.67]	0.44	1.00
	Hispanic Male	1.25 [0.74 to 2.11]	0.40	1.00
	White Male	1 [Reference]		
	18 to 44	0.91 [0.44 to 1.88]	0.80	
	45 to 54	1.21 [0.70 to 2.10]	0.48	
	55 to 64	1.68 [1.13 to 2.50]	0.01	
	65 to 74	1 [Reference]		
	75 or older	0.64 [0.44 to 0.95]	0.02	
**Accessing the internet at home**	**Need-Without-Risk**			
	Black Female	1.84 [0.78 to 4.35]	0.17	1.00
	Hispanic Female	2.64 [1.03 to 6.79]	0.04	0.43
	White Female	0.80 [0.37 to 1.72]	0.57	1.00
	Black Male	1.55 [0.85 to 2.81]	0.15	1.00
	Hispanic Male	1.36 [0.69 to 2.69]	0.37	1.00
	White Male	1 [Reference]		
	18 to 44	0.28 [0.12 to 0.68]	0.005	
	45 to 54	0.51 [0.18 to 1.50]	0.22	
	55 to 64	1.19 [0.55 to 2.58]	0.65	
	65 to 74	1 [Reference]		
	75 or older	1.28 [0.66 to 2.49]	0.46	
	**Risk-Without-Need**			
	Black Female	0.82 [0.57 to 1.17]	0.28	1.00
	Hispanic Female	0.90 [0.52 to 1.56]	0.71	1.00
	White Female	0.95 [0.66 to 1.36]	0.77	1.00
	Black Male	1.14 [0.88 to 1.48]	0.31	1.00
	Hispanic Male	1.35 [0.90 to 2.03]	0.15	1.00
	White Male	1 [Reference]		
	18 to 44	1.09 [0.66 to 1.82]	0.73	
	45 to 54	0.95 [0.64 to 1.41]	0.78	
	55 to 64	0.88 [0.66 to 1.17]	0.39	
	65 to 74	1 [Reference]		

© 2026 Russell LE et al. *JAMA Network Open*.

	75 or older	1.12 [0.89 to 1.42]	0.33	
**Assistance with legal issues**	**Need-Without-Risk**			
	Black Female	2.41 [1.10 to 5.27]	0.03	0.25
	Hispanic Female	1.98 [0.89 to 4.42]	0.10	0.58
	White Female	0.41 [0.17 to 0.98]	0.05	0.32
	Black Male	1.52 [0.81 to 2.85]	0.19	0.59
	Hispanic Male	1.98 [1.06 to 3.71]	0.03	0.25
	White Male	1 [Reference]		
	18 to 44	0.34 [0.16 to 0.75]	0.007	
	45 to 54	1.19 [0.43 to 3.29]	0.74	
	55 to 64	1.41 [0.70 to 2.82]	0.34	
	65 to 74	1 [Reference]		
	75 or older	1.03 [0.59 to 1.82]	0.91	
	**Risk-Without-Need**			
	Black Female	0.80 [0.35 to 1.83]	0.59	1.00
	Hispanic Female	0.42 [0.14 to 1.24]	0.12	0.59
	White Female	0.50 [0.21 to 1.22]	0.13	0.59
	Black Male	1.00 [0.60 to 1.69]	0.99	1.00
	Hispanic Male	0.36 [0.17 to 0.78]	0.009	0.09
	White Male	1 [Reference]		
	18 to 44	1.23 [0.40 to 3.72]	0.72	
	45 to 54	0.34 [0.12 to 0.95]	0.04	
	55 to 64	0.96 [0.52 to 1.76]	0.89	
	65 to 74	1 [Reference]		
	75 or older	0.74 [0.45 to 1.23]	0.25	
**Transportation for basic needs**	**Need-Without-Risk**			
	Black Female	1.45 [0.70 to 3.00]	0.31	1.00
	Hispanic Female	1.15 [0.44 to 2.98]	0.77	1.00
	White Female	0.43 [0.19 to 1.00]	0.05	0.41
	Black Male	2.36 [1.24 to 4.51]	0.009	0.09
	Hispanic Male	1.26 [0.61 to 2.60]	0.53	1.00
	White Male	1 [Reference]		
	18 to 44	1.90 [0.73 to 4.96]	0.19	
	45 to 54	0.49 [0.24 to 1.02]	0.06	
	55 to 64	1.05 [0.59 to 1.87]	0.86	
	65 to 74	1 [Reference]		
	75 or older	0.90 [0.52 to 1.57]	0.71	
	**Risk-Without-Need**			
	Black Female	1.19 [0.67 to 2.11]	0.56	1.00
	Hispanic Female	2.37 [1.02 to 5.51]	0.04	0.40

© 2026 Russell LE et al. *JAMA Network Open*.

	White Female	0.81 [0.44 to 1.49]	0.50	1.00
	Black Male	1.26 [0.77 to 2.07]	0.35	1.00
	Hispanic Male	1.09 [0.68 to 1.75]	0.72	1.00
	White Male	1 [Reference]		
	18 to 44	0.66 [0.34 to 1.28]	0.22	
	45 to 54	1.32 [0.60 to 2.92]	0.49	
	55 to 64	1.23 [0.69 to 2.19]	0.47	
	65 to 74	1 [Reference]		
	75 or older	1.07 [0.68 to 1.69]	0.75	
**Adult caregiving for self or others**	**Need-Without-Risk**			
	Black Female	1.45 [0.84 to 2.49]	0.18	1.00
	Hispanic Female	1.66 [0.78 to 3.52]	0.19	1.00
	White Female	0.99 [0.56 to 1.75]	0.98	1.00
	Black Male	2.13 [1.30 to 3.48]	0.003	0.03
	Hispanic Male	1.71 [1.05 to 2.77]	0.03	0.28
	White Male	1 [Reference]		
	18 to 44	0.66 [0.24 to 1.79]	0.41	
	45 to 54	0.47 [0.25 to 0.87]	0.02	
	55 to 64	0.89 [0.54 to 1.47]	0.66	
	65 to 74	1 [Reference]		
	75 or older	1.18 [0.77 to 1.80]	0.45	
	**Risk-Without-Need**			
	Black Female	0.80 [0.47 to 1.35]	0.40	1.00
	Hispanic Female	0.72 [0.36 to 1.44]	0.36	1.00
	White Female	0.69 [0.40 to 1.19]	0.18	1.00
	Black Male	1.14 [0.75 to 1.73]	0.53	1.00
	Hispanic Male	1.48 [0.74 to 2.96]	0.26	1.00
	White Male	1 [Reference]		
	18 to 44	0.82 [0.35 to 1.93]	0.66	
	45 to 54	0.79 [0.39 to 1.60]	0.51	
	55 to 64	1.03 [0.63 to 1.68]	0.92	
	65 to 74	1 [Reference]		
	75 or older	1.01 [0.68 to 1.50]	0.95	
**Getting additional education or job training**	**Need-Without-Risk**			
	Black Female	1.37 [0.37 to 5.07]	0.64	1.00
	Hispanic Female	0.48 [0.10 to 2.17]	0.34	1.00
	White Female	0.70 [0.17 to 2.95]	0.63	1.00
	Black Male	1.32 [0.49 to 3.61]	0.58	1.00
	Hispanic Male	0.70 [0.16 to 3.09]	0.64	1.00
	White Male	1 [Reference]		

© 2026 Russell LE et al. *JAMA Network Open*.

	18 to 44	12.90 [3.76 to 44.25]	<0.001	
	45 to 54	3.21 [1.46 to 7.03]	0.004	
	55 to 64	2.11 [1.05 to 4.24]	0.04	
	65 to 74	1 [Reference]		
	75 or older	0.78 [0.32 to 1.90]	0.58	
	**Risk-Without-Need**			
	Black Female	1.01 [0.64 to 1.60]	0.96	1.00
	Hispanic Female	1.27 [0.70 to 2.31]	0.43	1.00
	White Female	0.80 [0.49 to 1.30]	0.37	1.00
	Black Male	1.13 [0.79 to 1.60]	0.51	1.00
	Hispanic Male	1.69 [1.01 to 2.83]	0.04	0.44
	White Male	1 [Reference]		
	18 to 44	2.30 [1.31 to 4.04]	0.004	
	45 to 54	1.21 [0.75 to 1.94]	0.43	
	55 to 64	1.31 [0.91 to 1.89]	0.14	
	65 to 74	1 [Reference]		
	75 or older	0.52 [0.37 to 0.74]	<0.001	
**Getting or maintaining housing**	**Need-Without-Risk**			
	Black Female	1.65 [0.98 to 2.76]	0.06	0.41
	Hispanic Female	2.33 [1.13 to 4.81]	0.02	0.19
	White Female	1.14 [0.60 to 2.17]	0.70	1.00
	Black Male	2.67 [1.59 to 4.48]	<0.001	0.002
	Hispanic Male	2.19 [1.06 to 4.51]	0.03	0.27
	White Male	1 [Reference]		
	18 to 44	2.04 [1.03 to 4.04]	0.04	
	45 to 54	1.42 [0.75 to 2.68]	0.28	
	55 to 64	1.31 [0.84 to 2.04]	0.23	
	65 to 74	1 [Reference]		
	75 or older	0.50 [0.30 to 0.84]	0.008	
	**Risk-Without-Need**			
	Black Female	0.94 [0.54 to 1.63]	0.81	1.00
	Hispanic Female	0.77 [0.39 to 1.51]	0.45	1.00
	White Female	0.74 [0.40 to 1.35]	0.33	1.00
	Black Male	1.42 [0.99 to 2.05]	0.06	0.41
	Hispanic Male	1.47 [0.88 to 2.47]	0.14	0.71
	White Male	1 [Reference]		
	18 to 44	1.70 [0.83 to 3.48]	0.15	
	45 to 54	1.38 [0.82 to 2.33]	0.23	
	55 to 64	1.00 [0.69 to 1.46]	0.99	
	65 to 74	1 [Reference]		

© 2026 Russell LE et al. *JAMA Network Open*.

	75 or older	0.99 [0.74 to 1.33]	0.96	
**Finding or keeping work**	**Need-Without-Risk**			
	Black Female	0.39 [0.16 to 0.95]	0.04	0.39
	Hispanic Female	1.06 [0.43 to 2.63]	0.90	1.00
	White Female	1.09 [0.36 to 3.32]	0.88	1.00
	Black Male	1.16 [0.52 to 2.58]	0.71	1.00
	Hispanic Male	1.55 [0.70 to 3.44]	0.28	1.00
	White Male	1 [Reference]		
	18 to 44	1.01 [0.41 to 2.47]	0.99	
	45 to 54	2.87 [1.16 to 7.10]	0.02	
	55 to 64	3.06 [1.30 to 7.19]	0.01	
	65 to 74	1 [Reference]		
	75 or older	0.28 [0.12 to 0.68]	0.005	
	**Risk-Without-Need**			
	Black Female	0.69 [0.40 to 1.20]	0.19	1.00
	Hispanic Female	0.83 [0.41 to 1.67]	0.60	1.00
	White Female	0.60 [0.33 to 1.08]	0.09	0.79
	Black Male	0.76 [0.47 to 1.22]	0.26	1.00
	Hispanic Male	1.57 [0.84 to 2.93]	0.15	1.00
	White Male	1 [Reference]		
	18 to 44	4.24 [2.09 to 8.61]	<0.001	
	45 to 54	4.30 [2.45 to 7.56]	<0.001	
	55 to 64	2.78 [1.71 to 4.51]	<0.001	
	65 to 74	1 [Reference]		
	75 or older	0.35 [0.18 to 0.67]	0.001	
**Obtaining childcare**	**Need-Without-Risk**			
	Black Female	1.68 [0.42 to 6.71]	0.46	1.00
	Hispanic Female	3.12 [0.90 to 10.78]	0.07	0.65
	White Female	1.13 [0.20 to 6.35]	0.89	1.00
	Black Male	1.56 [0.48 to 5.09]	0.46	1.00
	Hispanic Male	2.86 [0.94 to 8.69]	0.06	0.63
	White Male	1 [Reference]		
	18 to 44	0.64 [0.25 to 1.63]	0.35	
	45 to 54	0.33 [0.08 to 1.35]	0.12	
	55 to 64	0.77 [0.19 to 3.06]	0.71	
	65 to 74	1 [Reference]		
	75 or older	0.19 [0.05 to 0.72]	0.01	
	**Risk-Without-Need**			
	Black Female	0.70 [0.27 to 1.83]	0.47	1.00
	Hispanic Female	0.90 [0.29 to 2.82]	0.86	1.00
	White Female	0.96 [0.34 to 2.66]	0.93	1.00

© 2026 Russell LE et al. *JAMA Network Open*.

	Black Male	1.65 [0.79 to 3.42]	0.18	1.00
	Hispanic Male	2.45 [0.63 to 9.59]	0.20	1.00
	White Male	1 [Reference]		
	18 to 44	2.13 [0.62 to 7.25]	0.23	
	45 to 54	1.07 [0.39 to 2.95]	0.89	
	55 to 64	1.55 [0.69 to 3.46]	0.29	
	65 to 74	1 [Reference]		
	75 or older	0.83 [0.35 to 1.95]	0.67	

Abbreviations: aRRR, adjusted relative risk ratio.

[a] A concordant response for the social risk and social need questions was used as the reference for the need-without-risk and risk-without-need discordant responses.
[b] *P* values were adjusted using Holm's method to control for family-wise error rate at 0.05.

© 2026 Russell LE et al. *JAMA Network Open*.
