# Supplementary material for: Racial, Ethnic, and Sex Differences in Social Risks and Social Needs Concordance Among Veterans
Source: JAMA Netw Open. 2026 Feb 17;9(2):e2559892. doi: 10.1001/jamanetworkopen.2025.59892 (PMC12914491; doi:10.1001/jamanetworkopen.2025.59892)
Supplement: Supplement 2. — Data Sharing Statement [file jamanetwopen-e2559892-s002.pdf]

Data Sharing Statement

Data
Data available: No

Additional Information
Explanation for why data not available: We will not make the data available due to VHA privacy concerns.
